# Improving Mechanical Properties of Mg-Al-RE Alloys with the Formed Dimples of Al_10_Mn_2_RE Particles and Activated Pyramidal <*a*> Slip with Mn Additions

**DOI:** 10.3390/ma16206747

**Published:** 2023-10-18

**Authors:** Jiandong Yang, Wuxiao Wang, Min Zhang, Jian Liu, Shaoyong Qin

**Affiliations:** 1 School of Material Science and Engineering, Xi’an University of Technology, Xi’an 710048, China; yangjiandong7c@163.com (J.Y.); qinshaoyong@xaut.edu.cn (S.Q.); 2 Xi′an Aeronautical Polytechnic Institute, Xi’an 710089, China; 3 Faculty of Printing, Packaging and Digital Media, Xi’an University of Technology, Xi’an 710054, China; liujian@xaut.edu.cn

**Keywords:** magnesium alloy, mechanical properties, fracture mechanism, dislocation slip, deformation twin

## Abstract

The effects of Mn addition on the room temperature tensile strength and deformation mechanisms of as-cast Mg-8Al-1Nd-1.5Gd-*x*Mn alloys (*x* = 0, 0.3, 0.5, 1.0 wt.%) are investigated in this paper. The results indicate that the addition of Mn contributes to the precipitation of Al-Mn-RE intermetallics and the refinement of *α*-Mg matrices, thereby improving the tensile strength of the 1.0 Mn alloy at 190 MPa. The fracture mechanism of Mn-containing alloys transforms from a cleavage fracture to a ductile fracture as the Mn content increases from 0.3 to 1.0 wt.%. The presence of intermetallic particles in the dimples confirms the hindrance effect of Al_10_Mn_2_ (Nd,Gd) on dislocation slips. The novel technology of in-grain misorientation axes (IGMAs) is used to identify activated slip modes and deformation twins. It can be concluded that the activated pyramidal *<a>* slip during tensile deformation significantly promotes the ductility of the 1.0 Mn alloy with an elongation rate of 9.8%. It is worth noting that reducing the coarse $\left\{10\bar{1}2\right\}$ tensile twins and enhancing the proportion of $\left\{10\bar{1}1\right\}$ compressive twins and $\left\{10\bar{1}1\right\}$-$\left\{10\bar{1}2\right\}$ double twins contributes to maintaining the continuous plastic deformation of Mg alloy.

## 1. Introduction

Mg-Al alloys have been extensively applied in the aerospace and automobile industries given their low density, excellent castability, and mechanical processing properties [1,2,3]. However, their mechanical properties and creep resistance deteriorate rapidly as their service temperature exceeds 120 °C because of softened and coarsened *β*-Mg_17_Al_12_ at high temperatures [4]. Mg-Al-RE-based alloys are applied to powertrain components in automobiles because of their excellent high-temperature creep resistance. This is mainly attributed to Al_2_RE and Al_11_RE_3_ intermetallics, the dominant strengthening phases, which are mainly distributed at grain boundaries and can effectively impede dislocation motion and grain boundary sliding [5,6,7,8]. The addition of rare earth (RE) elements can significantly enhance the tensile strength and creep resistance of these alloys while also sacrificing elongation [9]. To achieve a balance between tensile strength and elongation, manganese (Mn) is widely used as a trace element additive in Mg-Al-RE alloys. Using grain refinement on as-cast Mn-containing magnesium alloys is an important way to improve their deforming behavior [10,11]. Simultaneously, the addition of Mn can promote the precipitation of Al-Mn-RE intermetallics, which mainly form Al_8_Mn_5_, Al_10_RE_2_Mn_7_, and Al_12_RE_2_Mn_5_ in Mg-Al-RE-Mn alloys [9,12,13,14]. Mn is prone to reacting with Al, Nd, and Gd elements and can form binary or ternary phases, thereby continuously consuming the concentration of Al and reducing the *β*-Mg_17_Al_12_ phase content. Mn-induced grain refinement and diverse intermetallic compounds enable Mg-Al-RE-Mn alloys to exhibit excellent comprehensive mechanical properties [15,16,17]. It is generally believed that the addition of Mn can improve the tensile strength and elongation of magnesium alloys, mainly because of fine-grain strengthening. In addition, adding a solid solution of Mn atoms to a magnesium matrix leads to a decrease in the c/a ratio, which helps to initiate prismatic *<a>* slip and thus improves the plastic deformation ability of Mg alloys. Mg alloys are mainly subject to basal *<a>* slip at ambient temperatures, causing poor plasticity. Consequently, the deformation twins are activated to coordinate the deformation of the *c*-axis and improve the plastic deformation ability.

Currently, the dominant method for distinguishing dislocation structures is based on the invisibility criterion, *g*⋅*b* = 0 [18]. In terms of the micro-mechanisms of tensile fractures, previous studies have indicated that transgranular fractures and intergranular fractures are the main failure modes of Mg-Al-based alloys [19]. Transgranular fractures are susceptible to propagating at $\left\{10\bar{1}1\right\}$ twin grain boundaries in failed specimens with twin planes as the main cleavage planes [20]. The promoting effect of Mn on prismatic slips is still controversial, and the effect of different deformation twins ($\left\{10\bar{1}2\right\}$ tensile twins, $\left\{10\bar{1}1\right\}$ compressive twins, and $\left\{10\bar{1}1\right\}$-$\left\{10\bar{1}2\right\}$ secondary twins) on the elongation of the alloys has not been accurately determined [21,22]. At present, the analysis of tensile fracture mechanisms is mainly based on the observation of the fracture surface of tensile specimens, which can be classified as cleavage fractures or ductile fractures from a macro perspective. Unfortunately, the analysis of micro-deformation mechanisms, especially in the characterization of activated slip systems and deformation twins, is still lacking. The novel technology of in-grain misorientation axes (IGMAs) is proposed to analyze the dominant deformation mechanisms of basal slips, prismatic slips, and pyramidal slips in deformed grains [23,24,25,26]. Compared with the direct observation of dislocation structures using two weak-beam dark fields (WBDFs), IGMAs are more efficient in identifying slip systems by utilizing the intensity distribution of specific crystal axis rotations (1.2–2°) caused by slipping [25,27].

In this work, as-cast Mg-8Al-1Nd-1.5Gd-*x*Mn (*x* = 0, 0.3, 0.5, 1.0 wt.%) alloys are designed to investigate the influence of RE (Nd, Gd) and Mn composite modification on the tensile fracture mechanisms of Mg-Al alloys. The microstructures and fracture morphology of the tensile failure specimens are systematically characterized, and the underlying deformation mechanisms are analyzed by identifying the activated dominant slip and twins. The results are of profound significance for balance strength and plasticity in Mg-Al-RE alloys.

## 2. Experimental Material and Procedures

The specific alloys were prepared with magnesium ingot (99.99 wt.%), aluminum ingot (99.99 wt.%), Mg-30 Gd, Mg-30 Nd, and Mg-15 Mn (wt.%) intermediate alloys (Shanxi Yinguang Huasheng Magnesium Industry Co., Ltd., Yuncheng, China) Magnesium and aluminum ingots were initially melted in a resistance furnace at 710 °C in a protective atmosphere of Ar, and then the intermediate alloys were added for 30 min of insulation until the alloy was completely melted. The chemical composition of the alloys was homogenized through mechanical stirring. The molten Mg alloys were directly poured into a steel mold (preheated at 150 °C for 12 h) at 710 °C to obtain circular ingots with diameters of 50 mm. The alloy compositions were confirmed via inductively coupled plasma optical emission spectroscopy (ICAP6300 Radial, Thermo Fisher Scientific, MA, USA) as shown in Table 1. The prepared Mg-8Al-1Nd-1.5Gd-*x*Mn (*x* = 0, 0.3, 0.5, 1.0 wt.%) alloys are abbreviated as Mn-free, 0.3 Mn, 0.5 Mn, and 1.0 Mn alloys.

**Figure 1:**
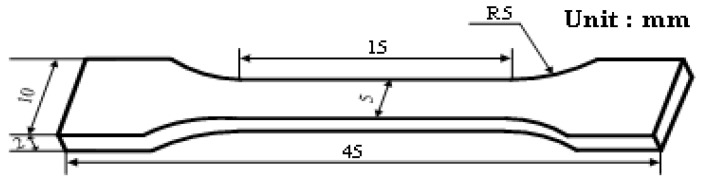
Schematic diagram of sample size for uniaxial tensile tests.

Tensile tests were conducted on a computer-controlled material testing machine with a tensile rate of 0.5 mm/min at ambient temperature. The average value of three tensile tests was taken for each specimen, as shown in Figure 1, and the deformation displacement was measured with an extensometer (the gauge length was 10.1 mm).

$$e=\frac{L_{K}-L_{0}}{L_{0}}\times 100\%$$

where e is percentage elongation, *L*_0_ is the original gauge length, and *L_K_* is the final gauge length after rupture.

The microstructure was characterized via X-ray diffraction (XRD) (SmartLab, Rigaku, Tokyo, Japan) with a scanning step of 5°/min and an SEM-6700F scanning electron microscope (SEM) equipped with an electron-backscattered diffraction (EBSD) and energy-dispersive spectrometer (EDS) (JEOL, Tokyo, Japan). The EBSD samples were electropolished with 10% perchloric acid ethanol electrolyte under a voltage of 30 V (10 °C) for 15 s, and then ion-polished at a voltage of 6 kV and an angle of 2°. The samples were polished at two points near and far away from the fracture, and each point was polished for 40 min. EBSD data were processed using Channel 5( Oxford Instruments, Abbington, Oxfordshire, UK) and slip systems and twins during tensile deformation were identified using IGMA technology. For TEM, the upper and lower surfaces of the samples were alternately thinned to allow for transparency at 3 kV, and FIB sample preparation was completed.

## 3. Results and Discussion

### 3.1. Microstructure of As-Cast Mg-8Al-1Nd-1.5Gd-xMn Alloys

**Figure 2:**
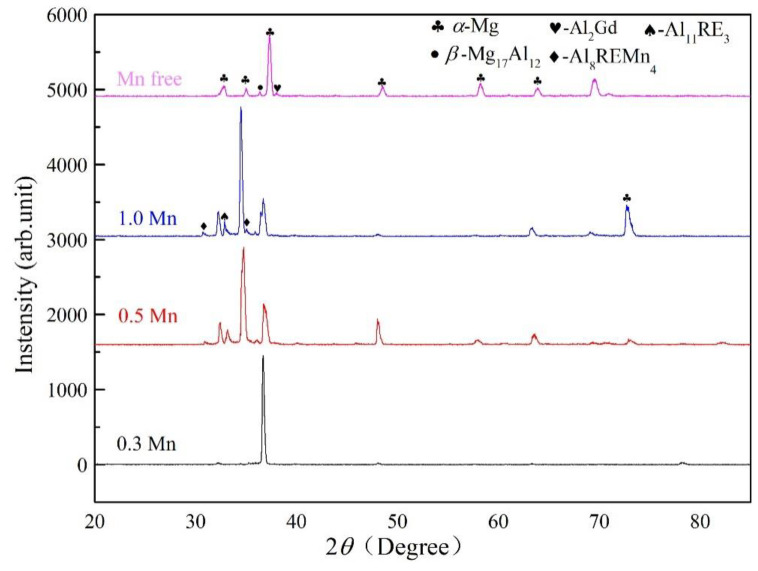
X-ray diffraction patterns of as-cast Mg-8.0Al-1.0Nd-1.5Gd-*x*Mn (wt.%) alloys.

**Figure 3:**
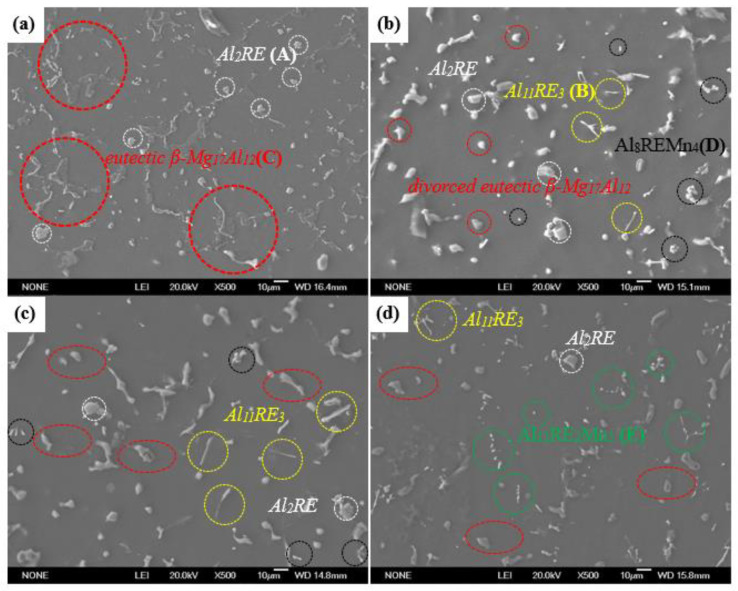
Typical SEM images of Mg-8Al-1Nd-1.5Gd-*x*Mn alloy*s*: (**a**) Mn-free, (**b**) 0.3 Mn, (**c**) 0.5 Mn, and (**d**) 1.0 Mn.

As shown in the typical XRD pattern of as-cast alloys in Figure 2, the studied alloys were composed of *α*-Mg, *β*-Mg_17_Al_12_, Al_2_RE, and Mn-containing intermetallic compounds combined with EDS (Table 2). The EDS results of points B and E exhibited a high amount of Mg, which might originate from the Mg matrix. There were no discernible diffraction peaks from the Al_8_REMn_4_ and Al_12_RE_2_Mn_5_ phases in the results, as firstly, the diffraction peaks of high-strength Mg caused the diffraction peaks of these two phases to be submerged, and secondly, the relatively low content of these two phases reduced the detection sensitivity. As shown in Figure 3a, massive, continuous island-like eutectic *β*-Mg_17_Al_12_ is distributed in the inter-dendritic region, and partial, completely divorced eutectic *β*-Mg_17_Al_12_ is scattered in the grains. In comparison with the Mn-free alloy, the volume fraction of intermetallic compounds obviously increased in the 0.3 Mn alloys (Figure 3b). Furthermore, the addition of 0.5 wt.% Mn transformed coarse eutectic *β*-Mg_17_Al_12_ into a granular divorced eutectic form while reducing the quantity of coarse Al_2_ (Nd,Gd) and increasing needle-shaped Al_11_ (Nd,Gd)_3_ (Figure 3c). Compared to the 0.5 Mn alloy, the 1.0 Mn alloy achieved a significant increase in Al-Mn-RE phases and a decrease in the content of eutectic *β*-Mg_17_Al_12_, as shown in Figure 3d. This is mainly due to the preferential precipitation of Mn-containing intermetallics at the solid–liquid front, which consumes a large amount of Al atoms and reduces the concentration of Al atoms involved in the formation of *β*-Mg_17_Al_12_, thereby reducing the volume fraction of the coarsened eutectic phase [28]. Compared with the Mn-free alloy, the Al_8_ (Nd,Gd)Mn_4_, Al_12_ (Nd,Gd)_2_Mn_5_, Al_11_ (Nd,Gd)_3_, and tiny *β*-Mg_17_Al_12_ phases shown in Mn-containing alloys replace the coarsened eutectic *β*-Mg_17_Al_12_ that deteriorates the mechanical properties of the alloy. Both granular Al_12_ (Nd,Gd)_2_Mn_5_ and needle-shaped Al_11_ (Nd,Gd)_3_ are beneficial for improving the strength of the alloy [29]. The preferentially solidified Al_2_ (Nd,Gd) and Al-Mn-RE phases are distributed at the solid–liquid front, hindering the growth of *α*-Mg matrix grains and ultimately refining their grains. 

### 3.2. Mechanical Properties of Mg-8Al-1Nd-1.5Gd-xMn Alloys

**Figure 4:**
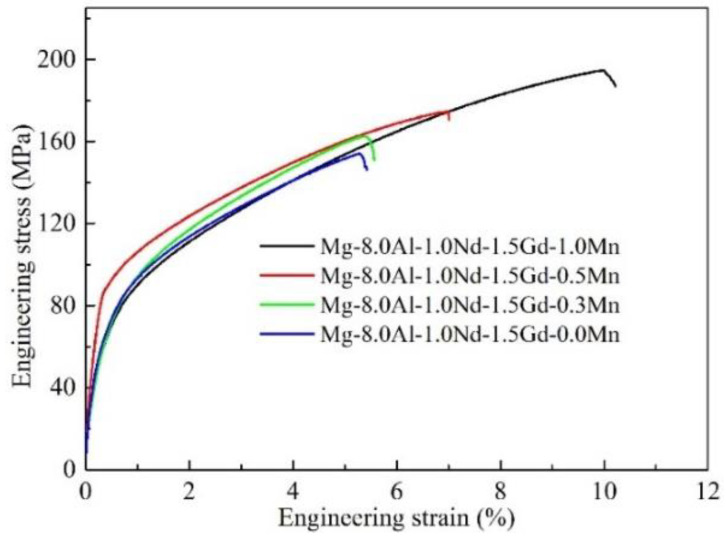
Tensile performance curves of the specific alloys.

The typical engineering stress–strain curves are illustrated in Figure 4, and the mechanical properties of Mg-8Al-1Nd-1.5Gd-*x*Mn alloys are illustrated in Table 3. The tensile tests of the as-cast alloys indicate a continuous increment in tensile strength and elongation as the Mn content increases. It is worth noting that the yield strength of the 0.5 Mn alloy was slightly higher, while there were no significant differences among the other alloys. This indicates that the addition of Mn had little effect on the yield strength of the alloy. However, compared to the yield strength, the tensile strength of the alloy varied significantly. The tensile strength and elongation increased from 89.9 to 110.9 MPa and from 5.1% to 9.8%, respectively. It can be concluded that the addition of Mn to Mg-8Al-1Nd-1.5Gd alloys improves their tensile strength and plastic deformation ability. Compared to the Mn-free alloy, the tensile strength and elongation of the 1.0 Mn alloy were increased by 25.8% and 92.2%, respectively. Meanwhile, the mechanical properties of various high-aluminum Mg alloys prepared with gravity casting are listed in Table 4. It can be seen that the 1.0 Mn alloy studied in this work exhibited excellent comprehensive mechanical properties, especially the elongation that reflects the plastic deformation ability of the alloy.

**Figure 5:**
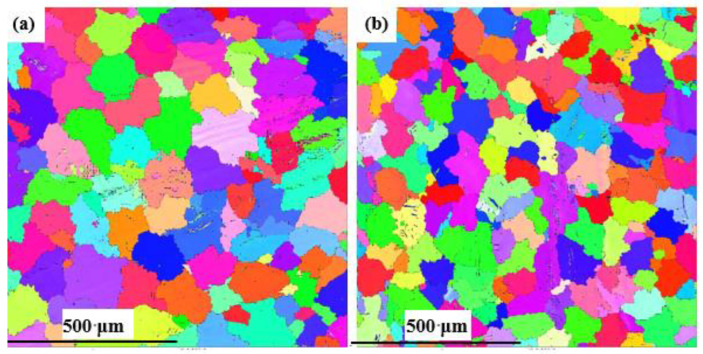
EBSD maps with grain boundaries of as-cast alloys: (**a**) Mg-8Al-1Nd-1.5Gd-0.3Mn; (**b**) Mg-8Al-1Nd-1.5Gd-1.0Mn.

To analyze the distinct mechanical properties of the alloys with different Mn content, the quantity, types, and grain size of precipitated intermetallics were also taken into account. The EBSD diagrams in Figure 5a,b illustrate that the average grain size of the *α*-Mg matrix in the 0.3 Mn alloy was 200 μm, while the corresponding grain size of the 1.0 Mn alloy was 152 μm. The particle size of intermetallic compounds in the 1.0 M alloy was the smallest, and the proportions of coarse Al_2_ (Nd,Gd) and Al_8_REMn_4_ phases were relatively low, thus exhibiting excellent mechanical properties. According to the Hall–Petch relationship (${∆\sigma }_{ys}=kd^{-1/2}$), where σ*_ys_* is the yield stress, *d* is the average grain size, and *k* is the stress concentration factor, the enhancement of tensile strength for Mn-containing alloy is closely related to grain refinement, which includes refinement of eutectic *β*-Mg_17_Al_12_ and *α*-Mg matrix grains [36]. In addition, various dispersed Al-Mn-RE (Al_12_RE_2_Mn_5_) and Al-RE (Al_11_RE_3_) intermetallic compounds effectively hinder the slip of dislocations and improve the tensile strength of the alloys [14,37]. To summarize, coarse eutectic *β*-Mg_17_Al_12_ in Mn-free alloys is the main factor that deteriorates the mechanical properties of the alloy. By adding Mn, the coarse eutectic *β*-Mg_17_Al_12_ is transformed into fine, granular, divorced eutectic structures. Although blocky Al_2_RE and Al_11_RE_3_ phases were detected in 0.3 Mn and 0.5 Mn alloys, the size and number of eutectic *β*-Mg_17_Al_12_ phases were significantly decreased. In addition, the precipitation of Al_11_RE_3_ had a positive effect on improving the tensile strength of the alloy.

### 3.3. Fracture Surface Characterization of Mg-8Al-1Nd-1.5Gd-xMn Alloys

**Figure 6:**
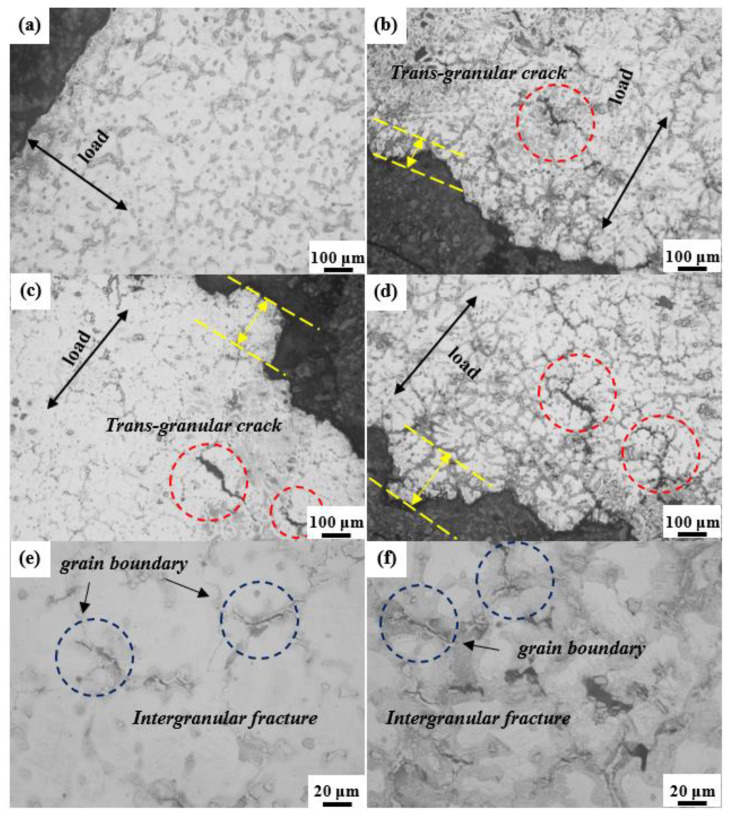
Longitudinal microstructures of fracture: (**a**) Mn-free; (**b**) 0.3 Mn; (**c**) 0.5 Mn; (**d**) 1.0 Mn, and (**e**,**f**) intergranular fracture in a failed specimen.

**Figure 7:**
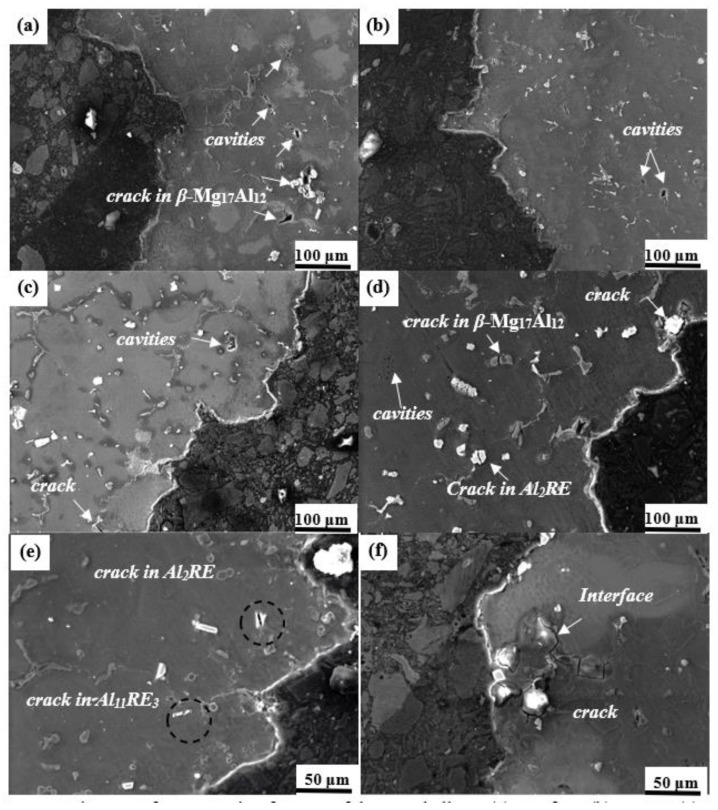
SEM images of cross-section fracture of the tested alloys: (**a**) Mn-free; (**b**) 0.3 Mn; (**c**) 0.5 Mn; (**d**) 1.0 Mn; (**e**) crack in Al_2_RE and Al_11_RE_3_; and (**f**) the propagation mode of the main crack.

Figure 6a–d show the metallographic images of the longitudinal tensile fracture surface of Mg-8Al-1Nd-1.5Gd-*x*Mn (*x* = 0, 0.3, 0.5, 1.0 wt.%) alloys. The fracture surface of the tensile specimens exhibited a typical serrated morphology, and the fluctuation height difference of the crack propagation path in the Mn-containing specimens was 120 to 210 μm (marked by yellow parallel lines). This indicates that more energy needs to be consumed during the dynamic fracture process [38]. The fracture surface of the Mn-free alloy is relatively flat, so the demand for energy during crack propagation is relatively low. Figure 6e,f show the macroscopic cracks in the tensile failure specimens initially forming and extending from the grain boundary. In addition, a small amount of trans-granular fracture mainly extended along the eutectic *β*-Mg_17_Al_12_ distribution direction, and the crack direction was perpendicular to the tensile direction. As shown in Figure 7a–d, the cavities were mainly concentrated in the fragmentation of coarse eutectic *β*-Mg_17_Al_12_ and aggregated to form microcracks [39]. Accordingly, reducing the content of eutectic *β*-Mg_17_Al_12_ and refining intermetallic compounds are effective means of improving mechanical properties. Figure 7e shows that the Al_2_RE and Al_11_RE_3_ are sheared into several parts under stress. Due to the high elastic moduli of these two phases and their excellent bonding strength with the *α*-Mg matrix, voids or fragments did not form, effectively preventing crack propagation. Figure 7f shows the propagation path of transgranular cracks in *α*-Mg, indicating that the main crack initially propagated from *β*-Mg_17_Al_12_ and propagated along the interface with the *α*-Mg matrix. This indicates that the eutectic *β*-Mg_17_Al_12_ is the main source of cracks. Al_2_RE and Al_11_RE_3_ can effectively suppress crack propagation, thereby improving the strength of the alloy.

**Figure 8:**
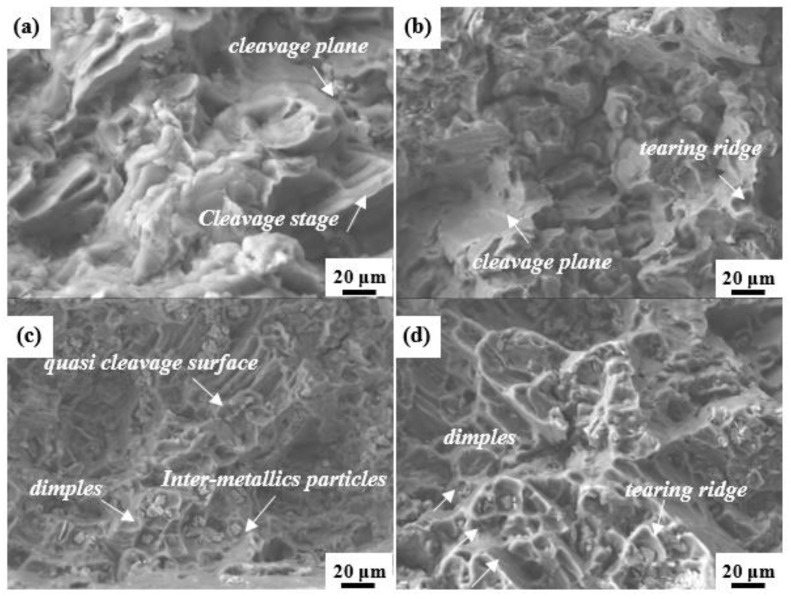
Microscopic fracture morphology of cracks: (**a**) Mn-free; (**b**) 0.3 Mn; (**c**) 0.5 Mn; (**d**) 1.0 Mn.

To further determine the fracture mechanism of the failed specimens, the enlarged fracture surface morphology is presented in Figure 8a–d. As shown in Figure 8a, the fracture surface of the Mn-free alloy is mainly composed of cleavage planes, exhibiting typical brittle fracture characteristics [40]. As the Mn content increased to 0.3 wt.%, the observed tearing edges indicated a transition from fracture to quasi-cleavage fracture (Figure 8b). Furthermore, the fracture morphology was composed of dimples and cleavage planes for the 0.5 Mn alloy, and the fracture behaviors were a combination of ductile and brittle fractures (Figure 8c). Compared to the 0.2 Mn alloy, the fracture surface of the 1.0 Mn alloy was covered with dense dimples, which were deeper and presented larger fluctuations in tearing edges (Figure 8d). In addition, the crack propagation became more tortuous, indicating the greater toughness of the 1.0 Mn alloy.

**Figure 9:**
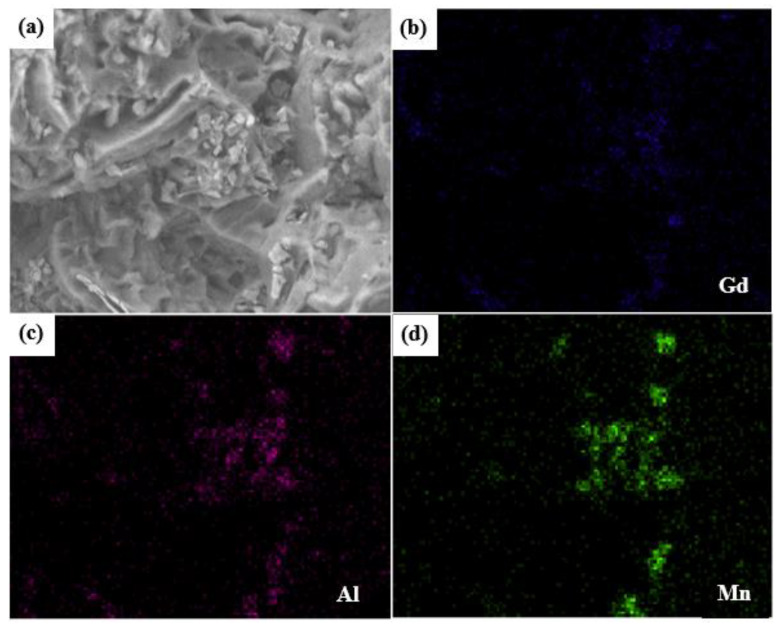
(**a**) Typical SEM backscattered image of the 1.0 Mn alloy with numerous intermetallic compound particles and element mapping of (**b**) Gd, (**c**) Al, and (**d**) Mn.

It is worth noting that intermetallic compound particles were observed in the dimples, and the size of the second-phase particles determines the size of the dimples. According to the energy spectrum of the 1.0 Mn alloy shown in Figure 9a–d, the particles in the dimples were mainly Al-Mn-RE phases, which can effectively hinder dislocation movement and promote the formation of dislocation accumulation, thereby improving the strength of the 1.0 Mn alloy [41,42]. 

It can be concluded that the coarse eutectic *β*-Mg_17_Al_12_ in the Mn-free alloy is the dominant factor leading to cleavage fracture. The strength of grain boundaries worsened due to *β*-Mg_17_Al_12_ during tensile deformation. With the addition of Mn, the coarse *β*-Mg_17_Al_12_ was replaced by Al-RE and Al-Mn-RE phases with high elastic moduli, resulting in dispersion strengthening. These dispersed particles effectively hinder dislocation slip during deformation, thereby alleviating stress concentration and coordinating grain deformation of the alloys [43]. This provides a reasonable explanation for the transition from cleavage fracture to ductile fracture.

**Figure 10:**
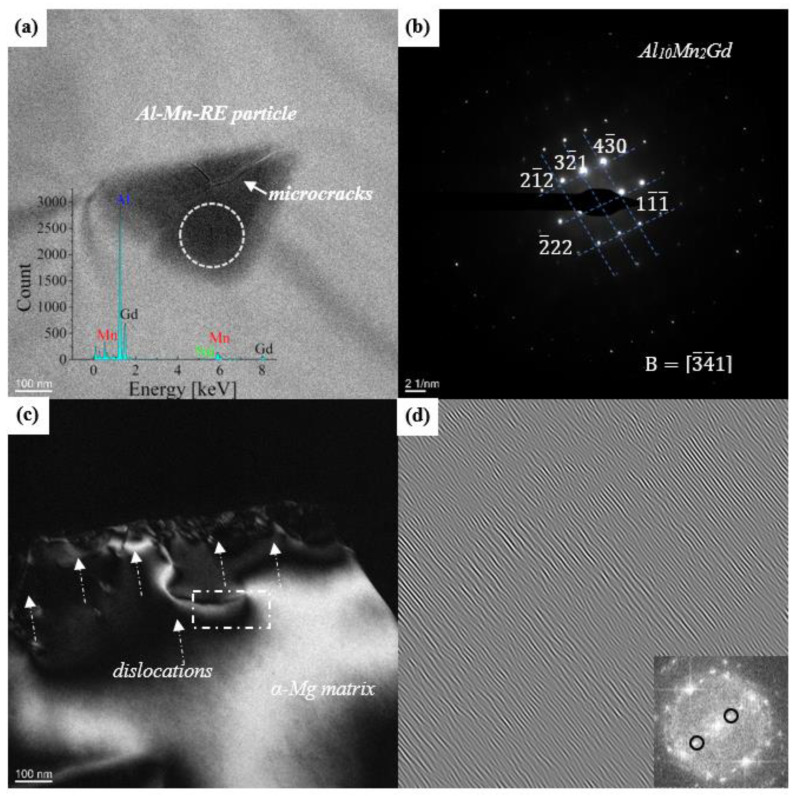
(**a**) BF-TEM micrograph and EDS of the Al-Mn-RE phase; (**b**) corresponding selected diffraction patterns of Al_10_Mn_2_Gd; (**c**) the dislocation morphology of the 1.0 Mn alloy with an elongation of 9.8%; and (**d**) the Fast Fourier transform of the dislocation region in the *α*-Mg matrix.

Figure 10a shows the representative BF-TEM image of the Al-Mn-RE phase; it can be observed that stress concentration caused the initiation of microcracks. According to the selected diffraction pattern in Figure 10b, the intermetallics can be identified as Al_10_Mn_2_RE. Moreover, the high-density dislocation near the fracture of the 1.0 Mn alloy is displayed in Figure 10c, and the high density of the edge dislocations can be seen in the crystal plane (Figure 10d) by performing a Fourier transform on dislocation regions, which further confirms that the sustained plastic deformation is related to dislocation slipping.

### 3.4. Analysis of Activated Twins and Slip Modes

**Figure 11:**
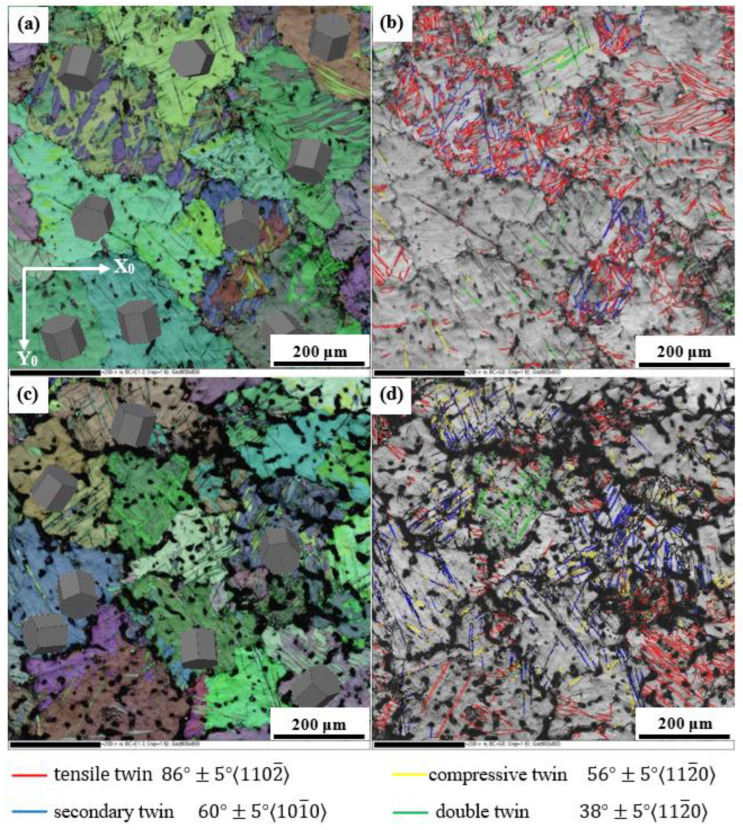
EBSD with all Euler and distribution of activated twins of tensile deformation structure: (**a**,**b**) 0.3 Mn alloy, (**c**,**d**) 1.0 Mn alloy.

To qualitatively identify the activated twins, in-grain misorientation axes (IGMA) analysis was performed on the tensile deformation of *α*-matrix grains. This method is based on twin-induced lattice rotation and the determination of its rotation axis [44]. As shown in Figure 11a, a large number of deformation twins were activated during the stretching process based on grain boundary character distribution. It has been reported that high-density twins exert an obvious grain refinement hardening effect in Mg-Al-Zn-Mn alloy [16]. The activated twins were dominated by $\langle 10\bar{1}2\rangle$ tensile twins and $\langle 10\bar{1}2\rangle$-$\left\{10\bar{1}2\right\}$ secondary twins, while small amounts of $\left\{10\bar{1}1\right\}$ compressive twins and $\left\{10\bar{1}1\right\}$-$\left\{10\bar{1}2\right\}$ double twins were detected in the 0.3 Mn alloy (Figure 11b). Compared to the 0.3 Mn alloy, the color gradient in the EBSD crystal orientation map of the 1.0 Mn alloy reflects a larger internal stress (Figure 11c), indicating that the alloy underwent more severe deformation during stretching. It is worth noting that the proportion of $\langle 10\bar{1}2\rangle$ tensile twins decreased, while the proportion of $\left\{10\bar{1}1\right\}$ compressive twins and secondary twins significantly increased (Figure 11d). The results illustrate that the activation of various twins is greatly affected by the concentration of Mn.

To quantitatively analyze the various twins shown in Figure 11, the statistical results are shown in Table 5. The total proportions of twins in the 0.3 Mn alloy and 1.0 Mn alloy were 23.85% and 23.4%, respectively. It has been confirmed that the $\left\{10\bar{1}2\right\}\langle \bar{1}011\rangle$ tensile twins are conducive to the plastic deformation of Mg alloys at room temperature [21,45]. In addition, the $\left\{10\bar{1}2\right\}$ twins are beneficial for activating the pyramidal II *<a+c>* slip and coordinating the deformation along the c-axis of the grain [22,46]. Furthermore, the interaction between lattice dislocation and the twin boundary plays a crucial role in the plastic deformation of Mg alloys [47]. The experimental results indicate that both the tensile and compressive twins were activated simultaneously, and the more uniform the distribution, the more favorable the plastic deformation of the alloys. Therefore, appropriately increasing the proportion of $\left\{10\bar{1}1\right\}$ twins and $60{}^{\circ}\pm 5{}^{\circ}\langle 10\bar{1}0\rangle$ secondary twins was beneficial to improving the tensile strength. This may be related to the grain refinement of the 1.0 Mn alloy, which leads to more uniform grain deformation under stress and contributes to suppressing crack initiation.

**Figure 12:**
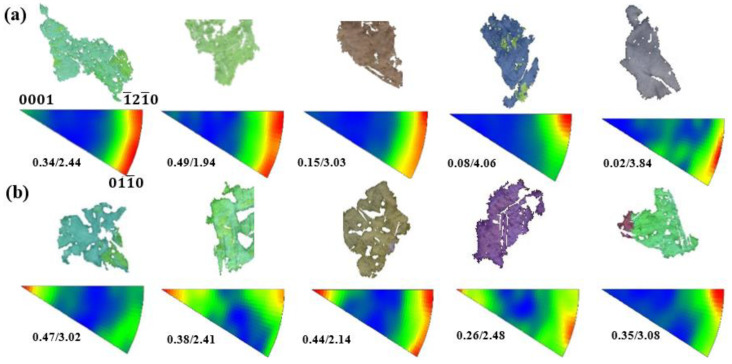
Deformed grains and corresponding IGMA distributions: (**a**) 0.3 Mn alloy; (**b**) 1.0 Mn alloy. The min/max intensities of the IGMA distribution are illustrated in the corresponding figure.

To further elucidate the deformation mechanism of a 1.0 Mn alloy with excellent plastic deformation ability, the IGMA method was implemented to identify the activated slip modes. The dominant slip system can be inferred in deformed grains by matching the Taylor axis (Table 6) [23,27]. As shown in Figure 12a,b, the results indicate that basal *<a>* and pyramidal *<a>* slip dominated in the 0.3 Mn alloy, while the proportion of prismatic *<a>* slip was very low, resulting in a weak distribution of intensities along the <0001> axis. As the Mn content increased to 1.0 wt.%, the prismatic *<a>* slip increased significantly. Accordingly, the basal *<a>* slip, accompanied by the prismatic *<a>* slip, which leads to a cross slip, is crucial for improving the plasticity of the alloy [48]. The addition of a solid solution of Mn atoms into the Mg matrix may lead to a change in the lattice parameter ***c*/*a*** ratio, thereby affecting the critical shear stress of the slip modes, which needs to be elaborated upon. The excellent elongation of the 1.0 Mn alloy is attributed to the significant activation of prismatic *<a>* slip and the increase in the proportion of compressive twins.

## 4. Conclusions

In this work, the influence of Mn modification on the mechanical properties of Mg-Al-RE alloys was discussed. The addition of Mn promotes the precipitation of various intermetallic compounds while promoting the activation of prismatic *<a>* slip, which can significantly improve the plastic deformation ability of the alloy. It is of great significance for improving the ductility of heat-resistant Mg-Al-RE alloys.

(1) The addition of Mn transforms continuous island-shaped eutectic *β*-Mg_17_Al_12_ into dispersed granular divorced eutectic particles and promotes the precipitation of Al_10_Mn_2_ (Nd,Gd) and Al_8_ (Nd,Gd)Mn_4_.
(2) The Al_10_Mn_2_ (Nd,Gd) particles can effectively hinder the slip of intragranular dislocations and alleviate stress concentration at grain boundaries. The dimples in the tensile fracture confirm the transition of the fracture mode from cleavage fracture to ductile fracture.
(3) The combination of activated prismatic *<a>* slip and deformation twinning enhances the plastic deformation ability of the Mg-Al-RE alloy.

## Figures and Tables

**Table 1:** Composition of designed alloys and corresponding values measured via ICP.

Alloys	Abbreviation	Elemental Composition (wt.%)				
		Al	Gd	Nd	Mn	Mg
Mg-8Al-1Nd-1.5Gd-0.0Mn	Mn-free	8.09	1.48	1.13	0.0	Bal.
Mg-8Al-1Nd-1.5Gd-0.3Mn	0.3 Mn	7.98	1.51	1.06	0.31	Bal.
Mg-8Al-1Nd-1.5Gd-0.5Mn	0.5 Mn	8.11	1.46	0.97	0.49	Bal.
Mg-8Al-1Nd-1.5Gd-1.0Mn	1.0 Mn	8.13	1.52	1.0	1.1	Bal.

**Table 2:** Measured compositions of various intermetallics in the 1.0 Mn alloy.

Isomorphous Phase	Composition (at. %)				
	Mg	Al	Nd	Gd	Mn
A (Al_2_RE)	13.83	61.13	9.50	14.21	1.33
B (Al_11_RE_3_)	57.43	32.93	4.0	5.15	0.54
C (*β*-Mg_17_Al_12_)	67.76	38.04	—	—	—
D (Al_8_REMn_4_)	9.54	55.90	2.56	5.91	26.08
E (Al_12_RE_2_Mn_5_)	57.59	16.55	1.17	0.95	5.74

**Table 3:** Mechanical properties of the alloys tested at room temperature.

Specimens	Tensile Strength/MPa	Elongation/%	Yield Strength/MPa
0.0 Mn	$154.2_{-1}^{+3}$	$5.1_{-0.2}^{+0.3}$	$101.4_{-2}^{+3}$
0.3 Mn	$162.2_{-1}^{+2}$	$5.4.2_{-0.1}^{+0.5}$	$104.2_{-1}^{+2}$
0.5 Mn	$174.5_{-2}^{+4}$	$6.9_{-0.2}^{+.06}$	$110.9_{-3}^{+5}$
1.0 Mn	$194.6_{-2}^{+5}$	$9.8_{-0.3}^{+0.74}$	$100.2_{-2}^{+4}$

**Table 4:** Tensile properties for gravity-die-cast Mg alloys.

Alloy (wt.%) Gravity Die Casting	Tensile Strength (MPa)	Yield Strength (MPa)	Elongation (%)	Ref.
AZ91	155~190	80~100	3.2~4.9	[30,31,32]
Mg-9Al-RE	165~187	84~116	4.0~6.4	
AZ80	160~182	95	5.1	[33,34,35]
AZ80-RE	182~191	90~110	5.6~7.9	
Mg-8Al-1Nd-1.5Gd-1.0Mn	199.6~192.6	104.2~98.2	9.5~10.5	

**Table 5:** Volume fraction of twin variants in tensile fracture specimens.

Activated Twins	Twinning Area Fractions	
	0.3 Mn Alloy (Strain 5.1%)	1.0 Mn Alloy (Strain 9.8%)
$\left\{10\bar{1}2\right\}$ tensile twins	20	10.8
$\left\{10\bar{1}1\right\}$–$\left\{10\bar{1}2\right\}$ double twins	1.5	5.5
$\left\{10\bar{1}1\right\}$ compressive twins	0.8	3.9
$\left\{10\bar{1}2\right\}$–$\left\{10\bar{1}2\right\}$ secondary twins	1.55.	3.2

**Table 6:** Activated slip modes in Mg and the corresponding Taylor axes.

Activated Slip	Deformation Mode	Variants	Taylor Axis
Basal *<a>*	$\left\{0001\right\}\langle 11\bar{2}0\rangle$	3	$\langle 01\bar{1}0\rangle$
Prismatic *<a>*	$\left\{10\bar{1}0\right\}\langle 11\bar{2}0\rangle$	3	$\langle 0001\rangle$
Pyramidal *<a>*	$\left\{10\bar{1}1\right\}\langle 11\bar{2}0\rangle$	6	$\langle 11\bar{2}0\rangle$
Pyramidal <*a+c*>	$\left\{11\bar{2}2\right\}\langle 11\bar{2}\bar{3}\rangle$	6	$\langle 1\bar{1}00\rangle$

## Data Availability

The data presented in this study are available on request from the corresponding author.
